# A Web-Based Computer-Tailored Program to Improve Treatment Adherence in Patients With Type 2 Diabetes: Randomized Controlled Trial

**DOI:** 10.2196/18524

**Published:** 2021-02-23

**Authors:** Stan Vluggen, Math Candel, Ciska Hoving, Nicolaas C Schaper, Hein de Vries

**Affiliations:** 1 Department of Health Promotion Maastricht University Maastricht Netherlands; 2 Department of Methodology and Statistics Maastricht University Maastricht Netherlands; 3 Department of Endocrinology and Internal Medicine Maastricht University Medical Centre Maastricht Netherlands

**Keywords:** type 2 diabetes mellitus, treatment adherence, eHealth, computer-tailoring, randomized controlled trial

## Abstract

**Background:**

Adherence to core type 2 diabetes mellitus (T2DM) treatment behaviors is suboptimal, and nonadherence is generally not limited to one treatment behavior. The internet holds promise for programs that aim to improve adherence. We developed a computer-tailored eHealth program for patients with T2DM to improve their treatment adherence, that is, adherence to both a healthy lifestyle and medical behaviors.

**Objective:**

The objective of this study is to examine the effectiveness of the eHealth program in a randomized controlled trial.

**Methods:**

Patients with T2DM were recruited by their health professionals and randomized into either the intervention group, that is, access to the eHealth program for 6 months, or a waiting-list control group. In total, 478 participants completed the baseline questionnaire, of which 234 gained access to the eHealth program. Of the 478 participants, 323 were male and 155 were female, the mean age was 60 years, and the participants had unfavorable BMI and HbA_1c_ levels on average. Outcome data were collected through web-based assessments on physical activity (PA) levels, caloric intake from unhealthy snacks, and adherence to oral hypoglycemic agents (OHAs) and insulin therapy. Changes to separate behaviors were standardized and summed into a composite change score representing changes in the overall treatment adherence. Further standardization of this composite change score yielded the primary outcome, which can be interpreted as Cohen *d* (effect size). Standardized change scores observed in separate behaviors acted as secondary outcomes. Mixed linear regression analyses were conducted to examine the effectiveness of the intervention on overall and separate treatment behavior adherence, accommodating relevant covariates and patient nesting.

**Results:**

After the 6-month follow-up assessment, 47.4% (111/234) of participants in the intervention group and 72.5% (177/244) of participants in the control group were retained. The overall treatment adherence improved significantly in the intervention group compared with the control group, reflected by a small effect size (*d*=0.27; 95% CI 0.032 to 0.509; *P*=.03). When considering changes in separate treatment behaviors, a significant decrease was observed only in caloric intake from unhealthy snacks in comparison with the control group (*d*=0.36; 95% CI 0.136 to 0.584; *P*=.002). For adherence to PA (*d*=−0.14; 95% CI −0.388 to 0.109; *P*=.27), OHAs (*d*=0.27; 95% CI −0.027 to 0.457; *P*=.08), and insulin therapy (*d*=0.35; 95% CI −0.066 to 0.773; *P*=.10), no significant changes were observed. These results from the unadjusted analyses were comparable with the results of the adjusted analyses, the per-protocol analyses, and the sensitivity analyses.

**Conclusions:**

Our multibehavior program significantly improved the overall treatment adherence compared with the control group. To further enhance the impact of the intervention in the personal, societal, and economic areas, a wide-scale implementation of our eHealth intervention is suggested.

**Trial Registration:**

Netherlands Trial Register NL664; https://www.trialregister.nl/trial/6664

## Introduction

Globally, 425 million people aged 20 to 79 years live with type 2 diabetes mellitus (T2DM), with expectations of over 600 million people being affected by 2045 [1]. T2DM is associated with considerable morbidity and mortality rates; it reduces patients’ quality of life and life expectancy and poses an enormous economic and societal burden [1,2]. Guidelines recommend a series of core treatment behaviors for patients with T2DM. These include healthy lifestyles, that is, improving dietary patterns and increasing physical activity (PA) and, if applicable, adequate adherence to medical strategies such as oral hypoglycemic agents (OHAs) whether or not combined with insulin therapy [3,4]. Despite the chronic progressive nature of T2DM, patients who adhere to these behaviors can live long, high-quality lives [1].

Unfortunately, patients’ adherence to separate behaviors is inadequate; dietary and PA targets are not met consistently, and most studies on adherence to medical strategies report adherence prevalence percentages below 80%, which is generally considered insufficient adherence [5-9]. Moreover, less than 5% of the patients diagnosed with T2DM adhere to all treatment behaviors, whereas more than 80% could either improve 2 or more [7]. Poor adherence can result in suboptimal clinical treatment benefits, such as disease worsening, an increase in comorbidity, a reduction in patients’ quality of life, increased health care expenditures and hospitalizations, and early mortality [2,10-16].

Nonadherence to core T2DM treatment behaviors such as healthy lifestyles and taking medication is a complex process and a result of an interaction of multiple factors, including social and economic factors, the health care system, characteristics of the disease and therapy, and patient-related factors [17,18]. Although all these factors provide relevant entries for targeting nonadherence, most are difficult to change, and if changed, they may only affect adherence improvements indirectly through patient factors [18]. However, patient-related factors (eg, awareness, beliefs, motivation, self-regulatory capacities) have been shown to be relatively changeable and have a direct impact on treatment adherence [19]. Hence, these determinants need to be addressed in interventions aimed at improving treatment adherence.

Several patient-focused interventions already exist that aim to improve treatment adherence. Most of these interventions pursued improvements in adherence to blood glucose–lowering medication [20-22], of which a minority showed significant improvements in medication adherence and glycemic control. Glycemic control is, however, not only the result of medication adherence but also greatly affected by (un)healthy lifestyle behaviors [18,23]. Therefore, interventions that target a combination of both healthy lifestyle and medical behaviors, that is, a multibehavior approach, might be more likely to be effective [7].

In addition to the multibehavior approach, other factors may enhance the effectiveness of interventions that aim to improve adherence. The internet holds promise for a wide-scale promotion of behavioral change to facilitate the management of T2DM [21,24,25]. Internet interventions as a delivery platform for health promotion and health service activities, also referred to as eHealth, have been shown to be effective, cost-effective, easy to use; have fewer availability restrictions than regular medical consultations; and can temper pressure on health care systems [26-32]. A more advanced eHealth strategy applies computer-tailored technology, an effective strategy that provides patients with tailored content based on unique answers given to a web-based assessment [33,34]. Further success factors of eHealth interventions include the application of a theoretical foundation; provision of interactive tailored content; application of goal-setting strategies and monitoring tools; identification of risk behaviors, using visually supported content; and focusing on distinct behavior change phases, that is, awareness, motivation, and self-regulation [21,24,25,35,36].

However, a recent review on eHealth interventions supporting T2DM management [25] concluded that only one of the 9 included studies reported significant improvements in dietary behavior and PA [36]. Generally, such eHealth interventions often include little interactive content and tailored strategies, are mainly text based, make little use of theoretical foundations and technology, and focus on separate behaviors that play a role in the management of T2DM instead of combining behaviors [21,24,25,35], which may explain the relatively poor results of available interventions.

Hence, eHealth interventions aimed at T2DM treatment adherence might be significantly improved by building on a theoretical base, incorporating computer-tailored technology, providing interactive and visually supported content, and applying a multibehavior approach.

Therefore, we developed an eHealth program for patients with T2DM, including the abovementioned success factors, to improve treatment adherence to core T2DM treatment behaviors, that is, healthy lifestyle and medical behaviors. The main aim of this study is to examine the effectiveness of this program on overall treatment adherence in a randomized controlled trial (RCT). In addition, we examined changes to separate treatment behaviors as a result of the program.

## Methods

### Study Design

We conducted an RCT including an intervention group and waiting-list control group to examine the effectiveness of a novel eHealth program, *My Diabetes Profile* (MDP), on treatment behavior adherence in patients with T2DM. A more extended description of the program, including its development and content and a trial protocol, is available elsewhere [37]. The study was evaluated and approved by the Medical Ethics Committee of Maastricht University Medical Centre (16-4-171). The committee concluded that no ethical clearance was needed according to the rules and regulations of the Medical Research Involving Human Subjects Act. The trial is registered in the Netherlands Trial Register (NL6664).

### MDP Program

The MDP program aims to improve patient adherence to core T2DM treatment behaviors. This implies improving PA levels; decreasing caloric intake from unhealthy snacks, as this emerged as a major issue in the diet of patients with T2DM in our preliminary work [38]; and increasing adherence to medical strategies, that is, OHAs whether or not combined with insulin therapy [3,4]. A screenshot of the main menu of the MDP program is presented in Multimedia Appendix 1.

The MDP program is theoretically grounded in the Integrated Change Model, which integrates various acknowledged sociocognitive theories that assume a deliberate process when someone engages in (health) behavior [39-43]. The model has frequently been applied to map salient sociocognitive determinants of health behavior and to develop effective web-based computer-tailored interventions aimed at health behavior change accordingly [44,45]. The MDP program is self-guided and facilitated through periodic prompts and reminders to stimulate program engagement and completion. The program provides web-based text and video feedback messages, tailored to determinants and underlying salient beliefs of health behavior change such as knowledge, attitudes, self-efficacy, goal setting, and action planning [43]. The program is divided into 2 nearly identical blocks, each available to users for 3 months. Each block consists of 3 sessions: (1) health risk appraisal; (2) awareness and motivation; and (3) goal setting, action planning, and self-regulation.

The health risk appraisal session provides patients with interactive and tailored content on their risk behaviors. Primarily, adherence levels are assessed for all behaviors the patient was involved in. For behaviors subject to improvement, the participants’ intention to change that behavior is assessed. The final part of the first session enables patients to select a single improvable behavior, which will be their focus for the following 3 months while working with the MDP program. In the event of meeting all guideline targets, patients are prompted to select PA as Dutch guidelines recommend any PA beyond the minimum weekly standard of 150 min [46]. A patient who selects a behavior that is accompanied by a low intention to change is navigated to the awareness and motivation session. This second session aims to raise patients’ awareness of the need to improve their particular behavior and to increase motivation, with the ultimate purpose of achieving a high intention to change. If a high intention to change is achieved, after either session 1 or session 2, the patient is directed to session 3 on goal setting, action planning, and self-regulation. This session aims to increase the likelihood of a successful translation of the expressed intention into subsequent behavior. This process is facilitated by setting small and realistic goals; forming action plans on where, when, and how to perform the behavior; and forming self-regulation strategies to cope with barriers or situations that may impede adherence.

### Participants and Procedure

In the Netherlands, patients usually visit their nurses every 3 months, under the supervision of a physician [3]. Therefore, these nurses were considered to be in an ideal position to recruit patients for this trial. Nationwide, nurses were approached via email, telephone calls, letters to their work address, and social media platforms (eg, LinkedIn and Facebook). They could sign up for the study by contacting the research team directly or by registering via the project website. Nurses were asked to recruit at least eight patients within a period of 6 months. Inclusion criteria for patients were (1) T2DM diagnosis for at least one year, (2) being 40 to 70 years old, (3) using at least one form of oral blood glucose–lowering drugs or insulin, and (4) having no walking disability. Exclusion criteria were (1) not speaking or understanding the Dutch language, (2) having no access to the internet, and (3) using an insulin pump.

After recruitment, nurses filled a brief web-based registration form consisting of the participant’s name, telephone number (optional), birth date, most recent HbA_1c_ level (a measure for glycemic control), the year of diabetes diagnosis, current diabetes medical strategy, and email address. Once registered, patients received an email, including log-in data, which primarily provided access to additional study and procedure information before providing informed consent. Participants would then fill the baseline questionnaire after which they were randomly allocated to either the intervention group (receiving program access for 6 months) or the control group (receiving care as usual). Individuals allocated to the control group were informed about their group allocation after baseline completion and notified that they would be invited for the follow-up assessment 6 months later. Moreover, they were informed about the possibility of accessing the MDP program after completing the follow-up assessment as part of the waiting-list control group. Randomization occurred at the individual level by means of computer software randomization. After randomization, nurses were able to review if their patients were allocated to the intervention or control group. For patients who received access to the MDP program, a brief summary of the patient’s activity and progress in the program was available to the particular nurse, which could voluntarily be discussed in subsequent face-to-face sessions [37].

### Measurements

The baseline questionnaire included 131 questions on demographic characteristics, comorbidities, smoking status, current PA levels, caloric intake from unhealthy snacks, and adherence to OHAs whether or not combined with insulin therapy. The questionnaires were identical for both trial groups.

#### Demographic Characteristics

Demographics assessed only at baseline included the participant’s gender (male or female), age, education level (low: no education up to lower technical education; medium: general secondary education up to secondary vocational education; or high: school of higher general secondary education up to university degree), body length, and nationality. Living arrangement (together or alone), net income (under or above average), work status (salaried or self-employed, no salaried employment, retired or disabled or incapacitated), T2DM medication type (oral blood glucose–lowering medication, insulin therapy, or a combination), and body weight were assessed at both baseline and follow-up. BMI was calculated as weight per length^2^.

#### Comorbidities

Questions on comorbidity assessed, at baseline only, whether participants were affected in the past or currently have conditions, including depression, stroke, heart failure, myocardial infarction, cancer, chronic obstructive pulmonary disease or asthma or bronchitis, rheumatoid arthritis or osteoarthritis, and Crohn disease.

#### PA

PA levels were assessed using the validated Short Questionnaire to Assess Health-Enhancing Physical Activity (SQUASH) [47]. SQUASH assesses various domains of PA, for which the average daily hours and minutes, and the number of days per week activities are carried out, are reported. Each domain corresponds to a specific metabolic equivalent of task (MET) value, an intensity and energy expenditure ratio of a task compared with energy expenditure while at rest [48]. As national guideline targets recommend at least moderate PA, that is, ≥3 MET, and because SQUASH includes 2 activities, that is, <3 MET, these activities were excluded [49]. The cumulative number of weekly PA minutes was calculated accordingly.

#### Unhealthy Snack Intake

Weekly caloric unhealthy snacks intake was assessed using a self-administered food frequency questionnaire (FFQ). The FFQ includes unhealthy snacks identified by earlier studies, complemented with snacks commonly consumed in the Netherlands [50,51]. The unhealthy snacks listed in the FFQ are translated into a particular amount of calories consumed, based on the calorie database of the Dutch nutrition center [52]. A total of weekly caloric intake was calculated based on the participant’s intake from unhealthy snacks.

#### OHA Adherence

Oral drug adherence was measured using the Probabilistic Medication Adherence Scale (ProMAS) questionnaire [53]. The scale includes 18 items that assess a variety of adherence behaviors. To reduce potential recall bias, a period of 3 months was added to every item [54]. This period was chosen because, in the Netherlands, most patients visit their nurse quarterly, and this visit comprises discussing treatment adherence and if pharmacological changes are needed [3]. A sum score was calculated for the 18 items, ranging from 0 to 18, with higher scores representing better adherence.

#### Insulin Therapy Adherence

Insulin therapy adherence was assessed through an adapted version of the ProMAS questionnaire and included 9 items that were assessed over a 3-month period. Nonrelevant items, that is, items that did not distinguish between adherence and nonadherence to insulin therapy were removed [37]. A sum score was calculated for the 9 items, ranging from 0 to 9, with higher scores representing better adherence.

### Primary Outcome, Primary End Point, and Power Calculation

The primary outcome was the composition score of changes in separate treatment behaviors addressed in the program. To create such a composition score, changes in each treatment behavior, that is, changes in PA levels, caloric intake from unhealthy snacks, and OHA and insulin therapy adherence, were standardized into separate change scores. For each treatment behavior in each participant, baseline scores were subtracted from the follow-up scores, yielding a change score. The change score for caloric intake was reversed as the program aimed to decrease caloric intake from unhealthy snacks. To standardize the outcomes of the different behaviors, given the varying units of measurement, the change score of each participant was divided by the pooled SD of the change scores of this specific behavior [55]. The pooled SD of the change scores of both trial groups was used. Finally, per participant, these separate standardized change scores were then summed into a composite change score [56].

The composite change score was transformed, that is, standardized further, to be interpreted as Cohen *d* (effect size), by dividing it for each participant by the pooled SD of these composite change scores. Again, the pooled SD of the composite change scores of both trial groups was used. The standardized composite change score is the primary outcome and Cohen *d* is the primary end point of this study.

The power calculation was based on the primary outcome. We aimed to detect a difference in the mean of the primary end point between trial groups of 0.4 in a two-tailed test at a 5% type I error rate [57]. Considering an intraclass correlation coefficient of 0.02 and a statistical power of 80%, 116 participants per trial group would be sufficient for the trial’s follow-up assessment [58]. Given an expected attrition rate of 50%, we aimed to include 464 participants with a completed baseline assessment.

### Secondary Outcomes: Changes in Separate Treatment Behaviors

In addition to calculating a standardized composite change score, separate changes in PA levels, caloric intake by unhealthy snacks, and OHA and insulin therapy adherence were calculated. A standardized change score per treatment behavior was calculated per participant by subtracting the baseline score from the follow-up score, yielding a change score. Subsequently, the change score of each participant was divided by the pooled SD of the change scores of this specific behavior, as described above [55]. The difference in the means of these standardized change scores between the intervention and control group can again be interpreted as Cohen *d,* indicating the effect size for separate treatment behaviors [57].

### Statistical Analyses

All analyses were conducted using SPSS version 24.0, with a 5% significance level. Frequency and descriptive analyses were used to describe the sample characteristics. Primary and secondary outcomes were analyzed according to the intention-to-treat principle. As participants were nested within nurses participating in the trial, linear mixed regression analyses were conducted to assess the effectiveness of the MDP program. Covariates in the model were included based on the assumptions of the theoretical framework that was applied in this study, and included gender, age, education level, net income, living arrangement, work status, BMI, HbA_1c_ level, T2DM medication type, recruitment nurse type, and depression status. Results from the unadjusted and adjusted analyses are presented for the primary and secondary outcomes.

Multiple imputation was used for missing values on covariates and outcome variables, which is valid under the assumption that values are missing at random [59,60]. In addition, sensitivity analyses were performed. These consisted of per-protocol analyses and imputation scenarios involving participants of whom the primary outcome was not available. For the per-protocol analyses, results of the unadjusted analyses were presented for the primary and secondary outcomes. For the imputation scenarios, 4 different imputation scenarios were performed for the primary outcome: 2 optimistic and 2 pessimistic scenarios. In the optimistic scenarios, we assumed that, compared with the per-protocol analysis, dropouts improved in the primary outcome, whereas in the pessimistic scenarios, we assumed that they deteriorated. For both the optimistic and pessimistic scenarios, 2 imputation variants were performed, as attrition was unequal in the groups of the trial, and this imbalance might have affected the results of the analysis. In the equal variant, the imputed value was drawn for both the intervention and control groups assuming a normal distribution of the outcome with a mean equal to the condition mean +/−1×SD. In the unequal variant, we used either a mean equal to the intervention mean +/−1×SD for the intervention group or a mean equal to the control mean +/−1.5×SD in the control group. This unequal variant reflects the possibility that the outcome on the average either improved or deteriorated to a lesser extent in the participants of the intervention group, compared with the control group. More effort was required from the participants of the intervention group, which may have affected attrition.

Logistic regression analysis was applied to examine selective attrition after randomization regarding background characteristics, including gender, age, education level, net income, living arrangement, work status, BMI, HbA_1c_ level, T2DM medication type, recruitment nurse type, depression status, and trial condition. In addition, we examined whether participants varied in their baseline adherence to treatment behaviors based on their retention status.

## Results

### Sample Characteristics

**Figure 1:**
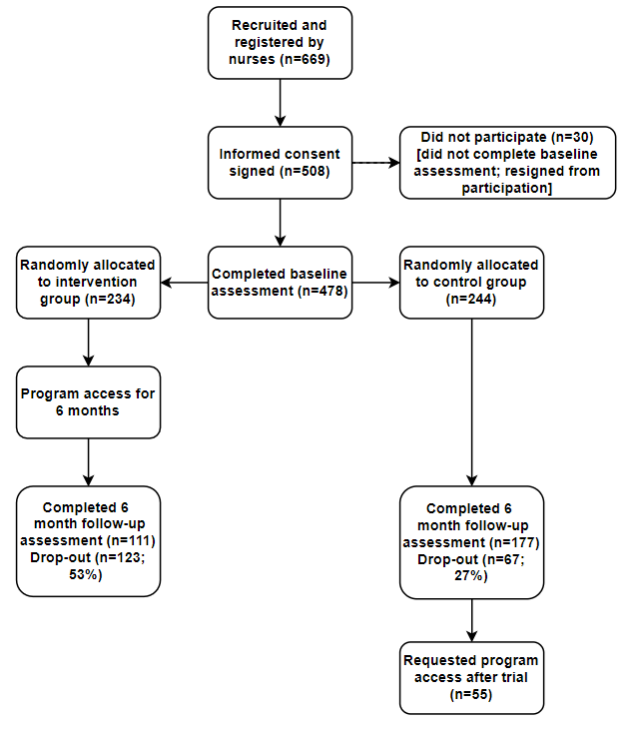
Participant and randomization flow throughout the trial.

Figure 1 shows the flow of participants throughout the trial from initial recruitment and registration by nurses to the completion of the follow-up assessment and requested program access by control group participants. In total, 669 participants were registered in the program. Overall, 75.9% (508/669) of participants signed the web-based informed consent. Overall, 94.1% (478/508) of participants completed the baseline assessment and were randomly allocated to either the intervention group (234/478, 48.9%) or control group (244/478, 51.0%).

The baseline sample of participants (Table 1) had a mean age of 60.2 (SD 6.78) years, approximately one-third were female, and 40.2% (192/478) were less educated. Most participants lived with their partners. A slight majority of the participants used OHAs as the only medication to control their blood glucose levels, whereas approximately one-third applied a combination of insulin and OHAs. The participants had unfavorable HbA_1c_ levels and BMI on average.

On average, patients could improve their three treatment behaviors. At the first occasion to select a single treatment behavior, most MDP participants chose to improve their PA levels (88/203, 43.3%), followed by decreasing unhealthy snacks intake (66/203, 32.5%), improving OHA adherence (39/203, 19.2%), and improving insulin therapy adherence (10/203, 4.9%). After 3 months, most participants chose to improve PA levels (46/104, 44.2%), followed by decreasing unhealthy snacks intake (39/104, 37.5%), improving OHA adherence (15/104, 14.4%), and improving insulin therapy adherence (4/104, 3.8%). Overall, 41.3% (43/104) of the participants chose the same behavior to improve on at the second occasion as at the start of the program. A total of 3 participants met all guideline targets, either on the first or on the second occasion, and were therefore prompted to select PA, as this was considered improvable regardless of the initial level [37].

The 6-month follow-up assessment was completed by 60.2% (288/478) of participants; 47.4% (111/234) in the intervention group and 72.5% (177/244) in the control group (Figure 1). Control participants were more likely to be retained in the study (OR 2.93, 95% CI 2.001 to 4.283; *P*<.001). Dropouts did not differ from those who were retained in terms of background characteristics and baseline adherence to treatment behaviors. About one-third of the control group participants requested program access, offered as part of the waiting-list control design, at the end of the study.

**Table 1:** Sample characteristics of patients at baseline and comparison of baseline characteristics between the intervention group (n=234) and control group (n=244).

Characteristic		Intervention group (n=234)	Control group (n=244)
Age in years, mean (SD)		60.9 (6.3)	59.4 (7.1)
Gender (female), n (%)		76 (32.5)	79 (32.4)
**Education level, n (%)**			
	Low	90 (38.5)	102 (41.8)
	Middle	56 (23.9)	52 (21.3)
	High	85 (36.3)	86 (35.2)
	Missing data	3 (1.3)	4 (1.9)
**Living arrangement, n (%)**			
	Together with partner	182 (77.8)	188 (77)
	Alone	52 (22.2)	55 (22.5)
	Missing data	N/A^a^	1 (0.4)
**Work status, n (%)**			
	Salaried or self-employed	96 (41.0)	111 (45.5)
	No salaried employment	35 (15.0)	39 (16.0)
	Retired	75 (32.0)	70 (28.7)
	Disabled or incapacitated	28 (12.0)	24 (9.8)
**Net income, n (%)**			
	Under-average income	60 (25.6)	70 (28.7)
	Above-average income	116 (49.6)	122 (50.0)
	Missing data	58 (24.8)	52 (21.3)
**Diabetes medication type, n (%)**			
	OHA^b^ only	143 (61.1)	151 (61.9)
	Insulin therapy only	11 (4.7)	21 (8.6)
	OHA and insulin therapy	80 (34.2)	72 (29.5)
**Recruitment nurse, n (%)**			
	Practice nurse	165 (70.5)	177 (72.5)
	Diabetes nurse	69 (29.5)	67 (27.5)
**Depression status, n (%)**			
	Never or in the past	224 (95.7)	228 (93.4)
	Current	10 (4.3)	16 (6.6)
HbA_1c_^c^ (mmol/mol), mean (SD)^d^		56.6 (11.8)	57.1 (11.8)
BMI (kg/m^2^), mean (SD)		30.8 (4.9)	31.2 (5.1)
OHA adherence score, mean (SD)		13.5 (3.5)	12.9 (4.0)
IT^e^ adherence score, mean (SD)		7.4 (1.8)	7.9 (1.6)
Physical activity (min), mean (SD)^f^		868 (1031)	764 (796)
Snack intake (cal), mean (SD)^f^		1746 (1435)	1676 (1375)

^a^N/A: not applicable.

^b^OHA: oral hypoglycemic agent.

^c^HbA_1c_: glycosylated hemoglobin.

^d^This equals an HbA_1c_ of 7.4%.

^e^IT: insulin therapy.

^f^Average number of weekly minutes or calories.

### Effect Analyses on Primary Outcome (Overall Treatment Adherence)

The results of the unadjusted and adjusted linear mixed regression analyses for the primary outcome are shown in Table 2. The result of the unadjusted analysis shows that allocation to the MDP program had a significant small effect on overall treatment adherence (*d*=0.27; 95% CI 0.032 to 0.509; *P*=.03). The adjusted result showed a similar small effect (*d*=0.25; 95% CI 0.010 to 0.495; *P*=.04). In total, 77.5% (86/111) of the participants in the intervention group showed improvement in overall treatment adherence compared with 60.5% (107/177) in the control group.

**Table 2:** Results of linear mixed regression analysis on multiple imputed data sets: unadjusted and adjusted model.

Linear mixed regression analysis		Regression coefficient (Cohen *d*)	95% CI	*t* test (*df*)	*P* value
**Unadjusted model (trial group)**					
	Intervention group	.27	0.032 to 0.509	2.229 (306.60)	.03
	Control group^a^	N/A^b^	N/A	N/A	N/A
**Adjusted model (trial group)**					
	Intervention group	0.25	0.010 to 0.495	2.05 (302.34)	.04
	Control group^a^	N/A	N/A	N/A	N/A
BMI		−0.01	−0.032 to 0.014	−0.78 (558.01)	.43
**Recruitment nurse**					
	Practice nurse	−0.05	−0.370 to 0.271	−0.31 (223.18)	.76
	Diabetes nurse^a^	N/A	N/A	N/A	N/A
**Diabetes medication type**					
	OHA^c^ only	0.01	−0.182 to 0.375	0.68 (454.77)	.50
	Insulin therapy only	−0.06	−0.572 to 0.451	−0.23 (288.59)	.82
	OHA and insulin therapy^a^	N/A	N/A	N/A	N/A
	HbA_1c_^d^	0.01	−0.005 to 0.019	1.19 (195.41)	.23
**Depression status**					
	Never or in the past	0.12	−0.650 to 0.407	−0.45 (307.75)	.65
	Current^a^	N/A	N/A	N/A	N/A
**Gender**					
	Male	−0.10	−0.381 to 0.174	−0.73 (259.33)	.46
	Female^a^	N/A	N/A	N/A	N/A
	Age (years)	−0.01	−0.030 to 0.016	−0.59 (354.14)	.56
**Work status**					
	Salaried or self-employed	−0.41	0.818 to 0.004	−1.95 (321.68)	.05
	No salaried employment	−0.56	−1.049 to −0.077	−2.28 (277.27)	.02
	Retired	−0.28	−0.733 to 0.167	−1.24 (319.11)	.22
	Disabled or incapacitated^a^	N/A	N/A	N/A	N/A
**Living arrangement**					
	Together with a partner	0.22	−0.082 to 0.522	−1.43 (291.29)	.15
	Alone^a^	N/A	N/A	N/A	N/A
**Education level**					
	Low	−0.37	−0.668 to −0.069	−2.42 (261.03)	.02
	Middle	−0.01	−0.308 to 0.288	−0.07 (602.33)	.95
	High^a^	N/A	N/A	N/A	N/A
**Net income**					
	Under-average income	0.28	−0.095 to 0.659	1.48 (147.14)	.14
	Above-average income^a^	N/A	N/A	N/A	N/A

^a^Reference category.

^b^N/A: not applicable.

^c^OHA: oral hypoglycemic agent.

^d^HbA_1c_: glycosylated hemoglobin.

### Effect Analyses on Secondary Outcomes (Separate Behavior Adherence)

The results of unadjusted linear mixed regression analyses for the secondary outcomes are shown in Table 3; the results of the adjusted analyses are presented in Multimedia Appendix 2. The results of the unadjusted analyses show that allocation to the MDP program had a significant small-to-medium effect on the decrease in caloric intake from unhealthy snacks (*d*=0.36; 95% CI 0.136 to 0.584; *P*=.002). The effects of oral hypoglycemic adherence (*d*=0.22; 95% CI −0.027 to 0.457; *P*=.08) and insulin therapy adherence (*d*=0.35; 95% CI −0.066 to 0.773; *P*=.10) were small but not significant. No effect was observed for PA (*d*=−0.14; 95% CI −0.388 to 0.109; *P*=.27). The results of the adjusted analyses of the secondary outcomes remained roughly equal to those of the unadjusted analyses.

**Table 3:** Results of the linear mixed regression analyses for separate treatment behaviors on multiple imputed data sets: unadjusted models.

Unadjusted models		Regression coefficient (Cohen *d*)	95% CI	*t* test (*df*)	*P* value
**OHA^a^ score**					
	Intervention group	0.22	−0.027 to 0.457	1.748 (338.47)	.08
	Control group^b^	N/A^c^	N/A	N/A	N/A
**IT^d^ score**					
	Intervention group	0.350.35	−0.066 to 0.773	1.658 (278.49)	.10
	Control group^b^	N/A	N/A	N/A	N/A
**PA^e^ level**					
	Intervention group	−0.14	−0.388 to 0.109	−1.107 (235.32)	.27
	Control group^b^	N/A	N/A	N/A	N/A
**Snack intake**					
	Intervention group	0.36	0.136 to 0.584	3.150 (474.10)	.002
	Control group^b^	N/A	N/A	N/A	N/A

^a^OHA: oral hypoglycemic agent.

^b^Reference category.

^c^N/A: not applicable.

^d^IT: insulin therapy.

^e^PA: physical activity.

### Sensitivity Analyses

Table 4 shows the adherence scores at baseline and follow-up per trial group and the results of the unadjusted per-protocol analyses. The effects on the primary and secondary outcomes were comparable with the intention-to-treat analyses. The results of the optimistic and pessimistic sensitivity analyses for the primary outcome partially reflected the results of the intention-to-treat and per-protocol analyses. After replicating the unadjusted linear mixed regression analyses following an equal and unequal optimistic imputation scenario, the intervention effect remained significant both under the equal (*d=*0.39; 95% CI 0.201 to 0.579; *P*<.001) and the unequal imputation (*d=*0.33; 95% CI 0.120 to 0.537; *P*=.002). Following an equal and unequal pessimistic imputation scenario, the intervention effect became nonsignificant under the equal (*d=*−0.13; 95 CI −0.353 to 0.090; *P*=.25) and unequal imputation (*d=*−0.04; 95% CI −0.271 to 0.189; *P*=.73).

In the intervention group, 73.9% (82/111) of the participants reduced their intake of unhealthy snacks compared with 54.2% (96/177) in the control group. One participant in the control group did not change in unhealthy snack score. With regard to oral blood glucose–lowering medication, 50.5% (52/103) of the intervention group participants improved their adherence compared with 42.9% (70/163) in the control group. 24.3% (25/103) of the participants in the intervention group did not change in OHA score compared with 20.9% (34/163) in the control group. With regard to insulin therapy adherence, 30.2% (13/43) of the participants in the intervention group improved their adherence compared with 27.9% (19/68) in the control group. Furthermore, 58.1% (25/43) of the participants in the intervention group did not change in insulin therapy score compared with 55.9% (38/68) in the control group.

**Table 4:** Adherence scores per trial group at baseline and follow-up, and the unadjusted per-protocol analyses for primary and secondary outcomes.

Treatment behavior	Adherence scores in the intervention group, mean (SD)			Adherence scores in the control group, mean (SD)			Cohen *d* (95% CI)	*t* test (*df*)	*P* value
	Baseline	Follow-up	n (%)	Baseline	Follow-up	n (%)			
Overall adherence	N/A^a^	N/A	111 (47.4)	N/A	N/A	177 (72.5)	0.24 (0.005 to 0.481)	2.006 (286)	.046
OHA^b^ score	13.4 (3.4)	14.3 (3.6)	103 (44.0)	13.1 (3.8)	13.4 (3.6)	163 (66.8)	0.18 (−0.070 to 0.423)	1.407 (264)	.16
IT^c^ score	7.4 (1.8)	8.0 (1.6)	43 (18.4)	7.7 (1.7)	7.9 (1.7)	68 (27.9)	0.28 (−0.102 to 0.658)	1.453 (109)	.15
PA^d^ level^e^	865 (1141)	833 (741)	111 (47.4)	789 (769)	884 (777)	177 (72.5)	−0.07 (−0.265 to 0.130)	−0.675 (286)	.50
Snack intake^f^	1857 (1330)	1269 (1182)	111 (47.4)	1656 (1331)	1496 (1108)	177 (72.5)	0.38 (0.098 to 0.661)	2.655 (286)	.009

^a^N/A: not applicable.

^b^OHA: oral hypoglycemic agent.

^c^IT: insulin therapy.

^d^PA: physical activity.

^e^Average number of minutes per week.

^f^Average number of calories per week.

## Discussion

### Principal Findings and Comparison With Previous Work

This study examined the effectiveness of a novel web-based, computer-tailored program, *MDP*, on overall adherence to core treatment behaviors in patients with T2DM. In addition, we explored the effects of the MDP program on each separate behavior. The MDP program improved overall adherence with a small, significant effect size. With regard to changes in adherence to each separate behavior, a small-to-medium significant effect size was observed for decreasing caloric intake from unhealthy snacks. Despite observing small-to-medium effect sizes for medication-taking behaviors, these showed no significance. With regard to adherence to PA, no significant changes were observed.

Our study focuses on improving adherence to multiple treatment behaviors in T2DM and is, to our knowledge, the first to subsequently quantify program effects in terms of the overall effect size. Generally, intervention studies put limited emphasis on exploring the overall change across risk behaviors [56]. Most studies focused on improving separate treatment behaviors, and the few targeting multiple risk behaviors have mainly examined changes in separate risk behaviors [24,25]. When risk behaviors co-occur, which is the case in the vast majority of patients with T2DM, the adverse effects on health and health outcomes are the largest [7,55,56]. Evaluating the effect of multibehavior interventions requires methods to quantify changes across several behaviors. Prochaska et al [55] supported quantifying the overall change in multiple behaviors by first calculating standardized change scores of each separate behavior and subsequently adding these scores [56,61]. However, although the application of such a composite change score has considerable advantages, it may be difficult to interpret as it is an abstract number. After further transforming this score, treatment effects can be interpreted as effect sizes (Cohen *d*), thereby increasing the interpretability of the observed results. Such an effect size also allows for comparison between distinct multibehavior interventions and examines the overall impact on health behavior change, which is not possible when focusing on changes in separate behaviors [56,62]. We recommend future studies to report multibehavior intervention effects in terms of effect sizes to improve interpretability and allow comparisons across studies.

Findings from this study appear to be robust and credible because the results of the unadjusted and adjusted intention-to-treat analyses were comparable with the per-protocol and sensitivity analyses. However, in the pessimistic missing data imputation scenarios, the main effect of the intervention became nonsignificant. Probably, the complete case scenario is the most accurate reflection of the actual intervention effect in this case because the attrition analysis revealed that differential attrition with regard to the demographic characteristics of the participants was absent [63-65].

When inspecting the nature of the overall effect and looking at the individual behaviors, we observed small-to-medium effect sizes for caloric intake from unhealthy snacks (*d*=0.36), insulin therapy adherence (*d*=0.35), OHA adherence (*d*=0.22), and a negligible effect size for PA (*d*=−0.14). Of these effects, only the effect for caloric intake was significant. Our results are in line with an earlier RCT in patients with T2DM on the effects of a web-based diabetes support program [37]. Directly after a 4-month intervention, significant improvements in healthy eating (*d*=0.32), fat intake (*d*=0.28), and PA (*d*=0.19) were observed in this trial; however, a negligible effect was observed for medication taking [36]. The effects in both our study and the aforementioned study may be related to specific success factors of eHealth interventions. Both interventions incorporated a theoretical foundation, interactive tailored content, and addressed multiple behaviors involved in the treatment of T2DM. Although it is difficult to examine the exact effect of such success factors, our findings support the conclusion that interventions applying sound theoretical motivational theories as a basis, interactive tailored content, and a multibehavior approach can have relevant effects [21,24,25,35,36].

A significant effect of our program was seen on eating behavior, that is, a decrease in caloric intake from unhealthy snacks. Improving dietary patterns in patients with T2DM is multifaceted, and generally, a decrease in fat intake is strived for [30,66]. Intake of unhealthy snacks recently emerged as a major novel issue in diets of patients with T2DM, according to both health professionals and patients themselves [38], and about a third of our participants selected this behavior to improve in the MDP program. Perhaps, the novelty of this diet topic and the detailed health risk appraisal complemented with personal feedback may have informed and alerted patients in such a way that they were triggered to successfully pursue a decrease in their caloric intake via snacks [67]. However, more research is needed to examine why a significant effect of our program occurred in caloric intake from unhealthy snacks.

No significant effects were observed for OHA and insulin therapy adherence and for PA. Observing no effects on PA levels seems to be common in digital multibehavior intervention studies [31,45,61,68-71]. However, PA was the behavior most often chosen by patients, and they were provided with a detailed health risk appraisal on their current PA levels. A more detailed analysis revealed that these patients had a high willingness to increase their PA and almost all could have chosen other topics to improve; therefore, they were not *forced* to pursue PA improvements. There are several potential explanations for the lack of any effect on the PA of our program. Patients could have overestimated their need and willingness to improve PA levels, as on average in this group, guideline targets were six-fold higher than the target of 150 min PA per week [72]. Note that these were self-reported values and were probably an overestimation, as several studies have shown that many persons are unable to reliably estimate their PA levels [73]. Using modern technology, such as accelerometers, this barrier might be overcome in the future [72]. It may also be that patients chose PA, as opposed to medication adherence improvements, as patients considered healthy lifestyle domains as more crucial to their health than medication taking [74,75]. Although improvements emerged in the other domains, that is, intake of unhealthy snacks, it might have been that improvements in this behavior required considerable self-control efforts. In turn, self-control spent on decreasing caloric intake may have depleted resources for further volitional efforts, such as improving or maintaining already high PA levels [76]. In fact, recent studies indeed show that high levels of self-control are required to translate short-term intentions into pursued PA improvements [77]. However, further research is needed to determine why multibehavior internet interventions seem to have such a limited effect on PA levels. For instance, reasons for failure could be explored in-depth using qualitative interviews, to analyze whether specific and effective action plans were made and whether PA plans were combined with other adherence activities that could have led to overdepletion in certain patients [76,78].

### Strengths and Limitations

Primarily, the multibehavior approach to improve treatment adherence is a strength of our study. Existing interventions have largely focused on improving single behaviors, whereas the management of T2DM is multifaceted and treatment nonadherence co-occurs across treatment behaviors. Second, the program was theory based and applied previously identified success factors for effective web-based self-management programs. Third, our nationwide recruitment and quite robust findings enhance the generalizability and credibility of our results. In fact, in a large Dutch survey study investigating characteristics of Dutch patients with T2DM, the distribution of education level, average age, living arrangement, paid employment, HbA_1c_ level, and BMI were comparable with the sample characteristics in our study [79]. In our study, slightly more people used T2DM medications, as this was a study requirement. Fourth, our study improved interpretability of the analysis results on the mean difference between intervention and control by transforming it into an effect size, which may also simplify comparisons of results of similar interventions in meta-analyses [57]. Moreover, changes in all behaviors were incorporated in the primary outcome, including changes to those behaviors that patients did not choose to receive feedback on in the intervention. Although this could potentially have reduced the effect identified, the overall score allowed us to correct our findings for changes in other behaviors (ie, improvements or compensation trade-offs) [74,80]. Finally, the attrition rate in our study was 53% in the intervention group and 27.5% in the control group. We did not observe differences between the participants who dropped out and those who did not; however, we cannot exclude that this might have affected our results. Moreover, the higher dropout rate in the intervention group could perhaps be a consequence of the necessary time investment. However, these results seem favorable as attrition rates reaching 60% to 80% are common in web-based interventions [81-83]; however, use of the program could probably be further stimulated by integrating it more in daily care. Feedback from noncompleters could yield valuable input to explore and improve retention rates and should therefore be addressed in future trials.

The limitations of our study are mainly methodological in nature. First, adherence data were collected through self-report questionnaires, which can be prone to social desirability issues and behavior overestimation [73,84]. To reduce the social desirability issue, future trials should consider objective measurement instruments for behaviors where this is possible, for example, accelerometers for PA and electronic monitor systems for medication adherence. However, the impact of such biases within this trial was likely reduced by applying baseline follow-up change scores and identical adherence assessments for participants in both trial groups. Second, we did not assess clinical outcomes such as glycemic control. However, as we did not include a postintervention follow-up period, it may be unlikely that such outcomes will be observed immediately after our intervention. Third, the nurses in our study were not blinded. For intervention group participants, nurses could voluntarily review and discuss the patient’s activity and progress in the program. However, it should be noted that nurses were not involved in the outcome assessment. Fourth, despite the advantages of a waiting-list control design that allows control participants to access the intervention after trial completion, some limitations apply such as a potential overestimation of treatment effects and delayed change by control group participants [85]. In our trial, however, the control group improved marginally in all outcomes, which may indicate a Hawthorne effect—the awareness of participants of being studied and the possible effect on behavior as a result—which is in accordance with trials not employing a waiting-list design [86]. Finally, we did not assess the long-term effects of our MDP program.

### Conclusions and Implications

The MDP program yielded larger improvements in overall treatment adherence postintervention, compared with our control group, reflected by a small overall effect size. Changes in separate behaviors yielded a significant small-to-medium effect size for decreasing caloric intake from unhealthy snacks, whereas small-to-medium but statistically nonsignificant effects were observed for medication-taking behaviors. Small-to-medium effect sizes, as observed in our study, may be of importance when multiplied to the population level [36], as the impact of the program depends not only on its effectiveness but also on its reach. To increase the reach of the MDP program, dissemination challenges could be explored, for example, if the current and scalable recruitment strategy is feasible in practice. Researchers could investigate health professionals’ willingness to adopt and implement the MDP program and whether reviewing the patient’s activity in the program is of added value to the professional. Implications for research include conducting a cost-effectiveness evaluation and a process evaluation. A process evaluation could yield information on the appreciation of the program, its working mechanisms, and adherence to the intervention and provide insights into the reasons for dropping out of the program. In addition, research could investigate long-term intervention effects and effects of the program on biomedical and societal outcomes such as glycemic control and quality of life. Further research could investigate the need for a more refined, composite score that may address the relative importance of different treatment elements in improving T2DM management. Finally, more research is needed to investigate how PA levels could be improved or maintained through eHealth interventions that aim for adherence improvements in multiple T2DM behaviors.

## Appendix

Multimedia Appendix 1 Main menu of the My Diabetes Profile program.

Multimedia Appendix 2 Adjusted secondary outcomes of the models after multiple imputations.

Multimedia Appendix 3 CONSORT-eHEALTH checklist (V 1.6.1).

## Abbreviations

FFQ: food frequency questionnaire
MDP: My Diabetes Profile
MET: metabolic equivalent of task
OHA: oral hypoglycemic agent
PA: physical activity
ProMAS: Probabilistic Medication Adherence Scale
RCT: randomized controlled trial
SQUASH: Short Questionnaire to Assess Health-Enhancing Physical Activity
T2DM: type 2 diabetes mellitus
