# Supplementary figures and images for: A Web-Based Computer-Tailored Program to Improve Treatment Adherence in Patients With Type 2 Diabetes: Randomized Controlled Trial
Source: J Med Internet Res. 2021 Feb 23;23(2):e18524. doi: 10.2196/18524 (PMC7943340; doi:10.2196/18524)

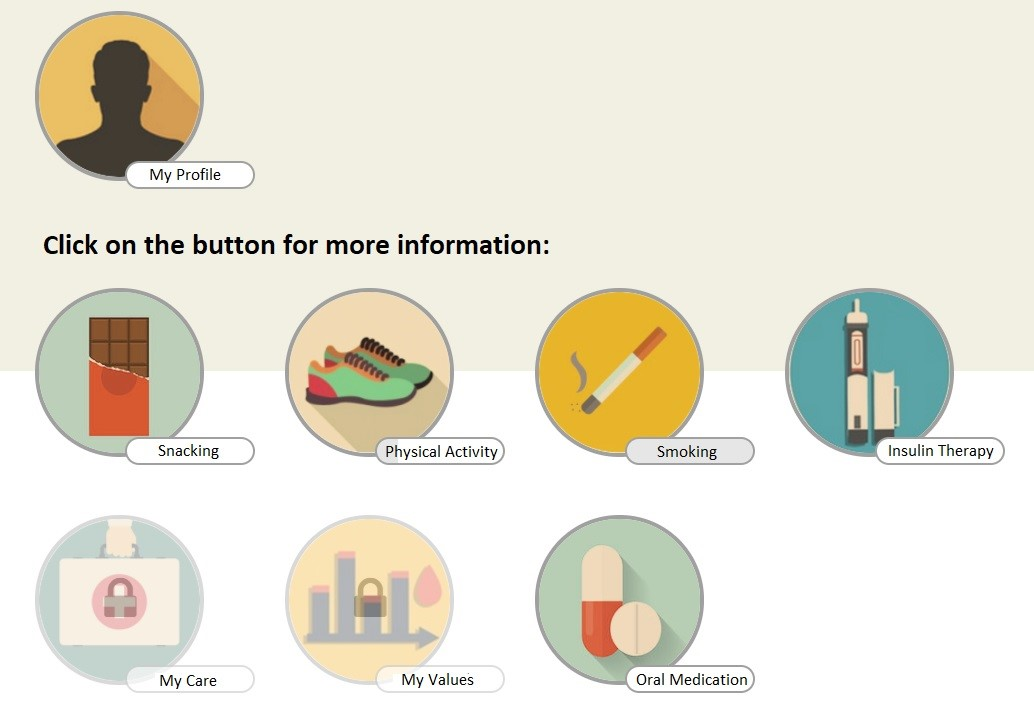

Supplement: Multimedia Appendix 1 [file jmir_v23i2e18524_app1.png]
