# Supplementary material for: A Web-Based Computer-Tailored Program to Improve Treatment Adherence in Patients With Type 2 Diabetes: Randomized Controlled Trial
Source: J Med Internet Res. 2021 Feb 23;23(2):e18524. doi: 10.2196/18524 (PMC7943340; doi:10.2196/18524)
Supplement: Multimedia Appendix 2 [file jmir_v23i2e18524_app2.docx]

| Fully adjusted model | Regression coefficient (Cohen’s *d*) | 95% Confidence Interval | T Value | *P* value |
| --- | --- | --- | --- | --- |
| **Trial arm**  Intervention arm  Control arm^a^  BMI  **Recruitment nurse**  Practice nurse  Diabetes nurse^a^  **Diabetes medication type**  OHA(s) only  OHA(s) and insulin therapy^a^  HbA_1c_  **Depression status**  Never/in the past  Current^a^  **Gender**  Male  Female^a^  Age  **Work status**  Salaried/Self employed  No salaried employment  Retired  Disabled/Incapacitated^a^  **Living arrangement**  Together with partner  Alone^a^  **Education level**  Low  Middle  High^a^  **Net. Income**  Under average income  Above average income^a^ | .23  .01  -.10  .11  .00  .18  -.22  -.02  -.50  -.45  -.42  .06  .04  -.07  -.19 | -.011 to .471  -.015 to .035  -.409 to .204  -.179 to .399  -.008 to .015  -.340 to .697  -.514 to .081  -.041 to .008  -.924 to -.085  -.918 to .023  -.899 to .056  -.268 to .390  -.286 to .364  -.391 to .246  -.610 to .240 | 1.88  -.79  -.66  .75  .56  .68  -1.43  -1.36  -2.36  -1.87  -1.74  .36  .24  -.45  -.87 | .06  .43  .51  .45  .57  .50  .15  .18  .02  .06  .08  .72  .81  .66  .39 |

### Supplementary table 1. Results of the full adjusted linear mixed regression analysis for oral hypoglycemic agent adherence on multiple imputed datasets

###

| Fully adjusted model | Regression coefficient (Cohen’s *d*) | 95% Confidence Interval | T Value | *P* value |
| --- | --- | --- | --- | --- |
| **Trial arm**  Intervention arm  Control arm^a^  BMI  **Recruitment nurse**  Practice nurse  Diabetes nurse^a^  **Diabetes medication type**  Insulin therapy only  OHA(s) and insulin therapy^a^  HbA_1c_  **Depression status**  Never/in the past  Current^a^  **Gender**  Male  Female^a^  Age  **Work status**  Salaried/Self employed  No salaried employment  Retired  Disabled/Incapacitated^a^  **Living arrangement**  Together with partner  Alone^a^  **Education level**  Low  Middle  High^a^  **Net. Income**  Under average income  Above average income^a^ | .39  -.04  -.05  -.02  .02  -.68  -.05  -.02  -.40  -.44  -.21  -.35  -.09  .12  -.20 | -.040 to .823  -.085 to -.002  -.438 to .342  -.551 to .507  .000 to .037  -1.827 to .467  -.534 to .429  -.062 to .018  -1.159 to .360  -1.309 to .432  -1.050 to .626  -.887 to .188  -.624 to .441  -.435 to .675  -.815 to .422 | 1.79  -2.07  -.24  -.08  1.98  -1.17  -.22  -1.09  -1.04  -.99  -.50  -1.28  -.34  -.43  -.63 | .08  .04  .81  .93  .05  .24  .83  .28  .30  .32  .62  .20  .74  .67  .53 |

### Supplementary table 2. Results of the full adjusted linear mixed regression analysis for insulin therapy adherence on multiple imputed datasets

###

### Supplementary table 3. Results of the full adjusted linear mixed regression analysis for caloric intake from unhealthy snacks on multiple imputed datasets

| Fully adjusted model | Regression coefficient (Cohen’s *d*) | 95% Confidence Interval | T Value | *P* value |
| --- | --- | --- | --- | --- |
| **Trial arm**  Intervention arm  Control arm^a^  BMI  **Recruitment nurse**  Practice nurse  Diabetes nurse^a^  **Diabetes medication type**  OHA(s) only  Insulin therapy only  OHA(s) and insulin therapy^a^  HbA_1c_  **Depression status**  Never/in the past  Current^a^  **Gender**  Male  Female^a^  Age  **Work status**  Salaried/Self employed  No salaried employment  Retired  Disabled/Incapacitated^a^  **Living arrangement**  Together with partner  Alone^a^  **Education level**  Low  Middle  High^a^  **Net. Income**  Under average income  Above average income^a^ | .33  .00  -.02  .27  -.06  .00  -.21  .03  .01  -.08  -.38  -.01  .42  -.39  -.05  -.53 | .109 to .555  -.021 to .027  -.339 to .290  -.001 to .538  -.580 to .466  -.010 to .016  -741 to .318  -.231 to .282  -.017 to .031  -.506 to .336  -.839 to .076  -.475 to .453  .132 to .702  -.706 to -.082  -.365 to .261  .212 to .848 | 2.93  .25  -.154  1.96  -.22  .48  -.79  -.20  .60  -.40  -1.64  -.05  2.88  -2.49  -.33  3.29 | .004  .81  .88  .05  .83  .63  .43  .85  .55  .69  .10  .96  .004  .01  .74  .001 |

### Supplementary table 4. Results of the full adjusted linear mixed regression analysis for physical activity on multiple imputed datasets

| Fully adjusted model | Regression coefficient (Cohen’s *d*) | 95% Confidence Interval | T Value | *P* value |
| --- | --- | --- | --- | --- |
| **Trial arm**  Intervention arm  Control arm^a^  BMI  **Recruitment nurse**  Practice nurse  Diabetes nurse^a^  **Diabetes medication type**  OHA(s) only  Insulin therapy only  OHA(s) and insulin therapy^a^  HbA_1c_  **Depression status**  Never/in the past  Current^a^  **Gender**  Male  Female^a^  Age  **Work status**  Salaried/Self employed  No salaried employment  Retired  Disabled/Incapacitated^a^  **Living arrangement**  Together with partner  Alone^a^  **Education level**  Low  Middle  High^a^  **Net. Income**  Under average income  Above average income^a^ | -.15  -.01  .08  -.00  .21  .00  -.00  -.05  .00  -.12  -.25  -.12  .11  -.22  .06  .19 | -.398 to .098  -.034 to .016  -.237 to .398  -.291 to .285  -.305 to .734  -.011 to .017  -.530 to .522  -.310 to .218  -.022 to .026  -.567 to .330  -.757 to .262  -.600 to .354  -.178 to .408  -.553 to .108  -.265 to .383  -.212 to .594 | -1.19  -.72  .50  -.02  .81  .47  -.01  -.34  .18  -.52  -.96  -.51  .77  -1.33  .36  .94 | .24  .47  .62  .98  .42  .64  .99  .73  .86  .60  .34  .61  .44  .19  .72  .35 |
